# Elevated Levels of the Complement Activation Product C4d in Bronchial Fluids for the Diagnosis of Lung Cancer

**DOI:** 10.1371/journal.pone.0119878

**Published:** 2015-03-23

**Authors:** Daniel Ajona, Cristina Razquin, Maria Dolores Pastor, Maria Jose Pajares, Javier Garcia, Felipe Cardenal, Michael Fleischhacker, Maria Dolores Lozano, Javier J. Zulueta, Bernd Schmidt, Ernest Nadal, Luis Paz-Ares, Luis M. Montuenga, Ruben Pio

**Affiliations:** 1 Program in Solid Tumors and Biomarkers, Center for Applied Medical Research (CIMA), Pamplona, Spain; 2 Laboratorio de Oncologia Molecular y Nuevas Terapias, Instituto de Biomedicina de Sevilla (IBiS), Hospital Universitario Virgen del Rocio/CSIC/Universidad de Sevilla, Sevilla, Spain; 3 Department of Histology and Pathology, School of Medicine, University of Navarra, Pamplona, Spain; 4 Department of Pulmonary Medicine, Clinica Universidad de Navarra, Pamplona, Spain; 5 Medical Oncology Department, Catalan Institute of Oncology-IDIBELL, Barcelona, Spain; 6 Molecular Biology Laboratory, Universitätsklinikum Halle, Saale, Germany; 7 Department of Pathology, Clinica Universidad de Navarra, Pamplona, Spain; 8 Department of Biochemistry and Genetics, School of Sciences, University of Navarra, Pamplona, Spain; University of Leicester, United Kingdom

## Abstract

Molecular markers in bronchial fluids may contribute to the diagnosis of lung cancer. We previously observed a significant increase of C4d-containing complement degradation fragments in bronchoalveolar lavage (BAL) supernatants from lung cancer patients in a cohort of 50 cases and 22 controls (CUN cohort). The present study was designed to determine the diagnostic performance of these complement fragments (hereinafter jointly referred as C4d) in bronchial fluids. C4d levels were determined in BAL supernatants from two independent cohorts: the CU cohort (25 cases and 26 controls) and the HUVR cohort (60 cases and 98 controls). A series of spontaneous sputum samples from 68 patients with lung cancer and 10 controls was also used (LCCCIO cohort). Total protein content, complement C4, complement C5a, and CYFRA 21-1 were also measured in all cohorts. C4d levels were significantly increased in BAL samples from lung cancer patients. The area under the ROC curve was 0.82 (95%CI = 0.71–0.94) and 0.67 (95%CI = 0.58–0.76) for the CU and HUVR cohorts, respectively. In addition, unlike the other markers, C4d levels in BAL samples were highly consistent across the CUN, CU and HUVR cohorts. Interestingly, C4d test markedly increased the sensitivity of bronchoscopy in the two cohorts in which cytological data were available (CUN and HUVR cohorts). Finally, in the LCCCIO cohort, C4d levels were higher in sputum supernatants from patients with lung cancer (area under the ROC curve: 0.7; 95%CI = 0.56–0.83). In conclusion, C4d is consistently elevated in bronchial fluids from lung cancer patients and may be used to improve the diagnosis of the disease.

## Introduction

Lung cancer is the leading cause of cancer-related death worldwide [1]. The overall five-year survival rate for lung cancer is approximately 15–20%, and less than 5% in metastatic cases [2]. One of the reasons for such a dismal outcome is the lack of effective techniques for early diagnosis of the disease. Currently, lung cancer diagnosis involves the combination of radiological and histological analyses of lesions. Flexible bronchoscopy represents a relatively noninvasive initial diagnostic test in individuals with suspected disease, and is the primary diagnostic tool in patients with centrally located lung cancer. Bronchoscopic techniques for the diagnosis of lung cancer include cytological examination of specimens from bronchial biopsy, bronchial brush, bronchial wash, and bronchoalveolar lavage (BAL) [3, 4]. Specificity of cytology of bronchoscopic material is 100%; however, sensitivity remains low, especially in more peripheral lesions, for which more invasive diagnostic procedures are routinely needed [5]. Thus, there is a clinical demand of adjunct markers that may improve the sensitivity of lung cancer diagnostic procedures.

Multiple biomarkers detectable in bronchial fluids from lung cancer patients have been proposed [6, 7, 8, 9, 10, 11, 12]. Recently, we have shown that lung cancer cells efficiently activate the classical pathway of complement. As a consequence, C4d, a stable split product of this pathway, is found elevated in plasma and BAL samples from lung cancer patients [13]. The aim of the present study was to validate our previous observation in independent case-control cohorts. We also aimed to compare the diagnostic performance of C4d with other potential diagnostic biomarkers: CYFRA 21-1, total protein, C4, and C5a. CYFRA 21-1 has long been proposed as a lung cancer biomarker in bronchial fluids [7, 14], plasma proteins are increased in BAL fluids from lung cancer patients [15], C4 is an abundant plasma protein from which C4d is generated after complement activation [16], and C5a is an active complement fragment increased in plasma samples from patients with non-small cell lung cancer [17]. Our results suggest that the determination of C4d in airway fluids outperforms the other markers and may be useful in the diagnostic workup of patients with lung cancer.

## Materials and Methods

### Clinical samples

The study included three cohorts of BAL samples and one of sputum specimens. BAL fluids were obtained from subjects undergoing diagnostic bronchoscopy and stored at −80°C. The procedure for BAL collection has been previously described [15]. The cohort from Clinica Universidad de Navarra (CUN) included BAL samples from 50 patients with lung malignancies and 22 patients with nonmalignant lung diseases. More details of this cohort have been previously reported [15]. The cohort from Charité-Universitätsmedizin (CU) included BAL specimens from 25 lung cancer patients and 26 control subjects. These control individuals underwent bronchoscopy for non-malignant airway diseases such as infection, benign airway stenosis or sarcoidosis. No additional data were available from this cohort. The third cohort was obtained at the Hospital Universitario Virgen del Rocio (HUVR) and included BAL fluids from 60 lung cancer patients and 98 control patients. The characteristics of these patients are shown in Table 1. Bronchoscopy was required in control patients due to the presence of hemoptysis or a pulmonary lesion in the chest-x ray or CT scan. Finally, the Lung Cancer Clinic of Catalan Institute of Oncology (LCCCIO) cohort of sputum specimens included 68 samples from lung cancer patients and 10 samples from healthy individuals. Samples were collected after spontaneous expectoration, diluted in 10 ml of saline, extensively vortexed, and stored at −80°C. All study protocols were performed according to the Declaration of Helsinki, were approved by the Research Ethics Committee of the University of Navarra (Institutional Review Board#015-2014), and all patients gave written informed consent. Lung tumors were classified using the WHO 2004 classification and the International System for Staging Lung Cancer [18, 19].

**Table 1:** Demographics and C4d levels in BAL fluids of patients from the HUVR cohort.

Characteristics		Lung cancer patients			Non-cancer patients	
	n	C4d (μg/ml) Median (IQR)	P^1^	n	C4d (μg/ml) Median (IQR)	P^1^
**Sex**						
Male	54	0.19 (0.15–0.26)	0.232	79	0.16 (0.14–0.18)	0.562
Female	6	0.15 (0.14–0.59)		19	0.15 (0.14–0.17)	
**Age** (years)						
≤60	21	0.17 (0.15–0.22)	0.329	46	0.15 (0.14–0.17)	0.300
>60	39	0.21 (0.15–0.31)		52	0.16 (0.15–0.18)	
**Smoking status**						
Former	34	0.21 (0.15–0.52)	0.065	59	0.16 (0.15–0.18)	0.058
Current	26	0.17 (0.15–0.21)		39	0.15 (0.14–0.16)	
**Pack-years**						
≤35	7	0.21 (0.17–0.27)	0.199	23	0.16 (0.15–0.18)	0.545
>35	37	0.18 (0.15–0.24)		32	0.15 (0.14–0.18)	
Not available	16	0.22 (0.16–0.72)		43	0.16 (0.14–0.17)	
**COPD**						
No				43	0.17 (0.15–0.18)	0.849
Yes				18	0.16 (0.15–0.19)	
Not available				37	0.15 (0.14–0.17)	
**Histology**						
AC	9	0.15 (0.14–0.17)	0.158			
SCC	28	0.20 (0.16–0.29)				
SCLC	13	0.22 (0.14–0.64)				
Other	7	0.20 (0.17–1.10)				
Not available	3	0.21 (0.19–0.74)				
**Stage**						
I-III	24	0.18 (0.15–0.25)	0.249			
IV	29	0.20 (0.16–0.53)				
Not available	7	0.16 (0.14–0.22)				

^1^Mann-Whitney U test, except for comparison of histologies (Kruskal-Wallis test).

### Marker measurements

C4d-containing fragments were measured by an enzyme-linked immunosorbent assay (Quidel). This assay recognizes all C4d-containing fragments of activated C4 (C4b, iC4b and/or C4d), which together are referred to in this paper as C4d. Quantitative enzyme-linked immunosorbent assays were also used for the determinations of C4 (Assaypro), C5a (R&D), and CYFRA 21-1 (DRG International). BAL samples were diluted 1:10, 1:1000, 1:100, and 1:25 for the analysis of C4d, C4, C5a, and CYFRA 21-1, respectively. Sputum specimens were diluted 1:4 for C4d quantitation. Total protein was measured using the BCA protein assay (Pierce). All biomarker measurements were performed retrospectively by laboratory personnel not aware of the diagnosis. All procedures were carried out in a single laboratory following the manufacturers’ instructions.

### Statistical analyses

SPSS 15.0 software was used for statistical analysis. Normality was assessed using Shapiro-Wilk test. Two-sided Mann-Whitney U test or Kruskal-Wallis H test were used to compare non-normally distributed data from two or more groups, respectively. Marker levels are shown as median (interquartile range). Receiver operating characteristic (ROC) curves were generated in order to evaluate the diagnostic performance of the biomarkers. Statistical differences between the area under the ROC curves and a reference area under the ROC curve of 0.5 were calculated with a z-score test. Ninety five percent confidence intervals were also calculated. P values less than 0.05 were considered statistically significant.

## Results

### C4d levels in BAL supernatants from lung cancer patients

**Fig 1:**
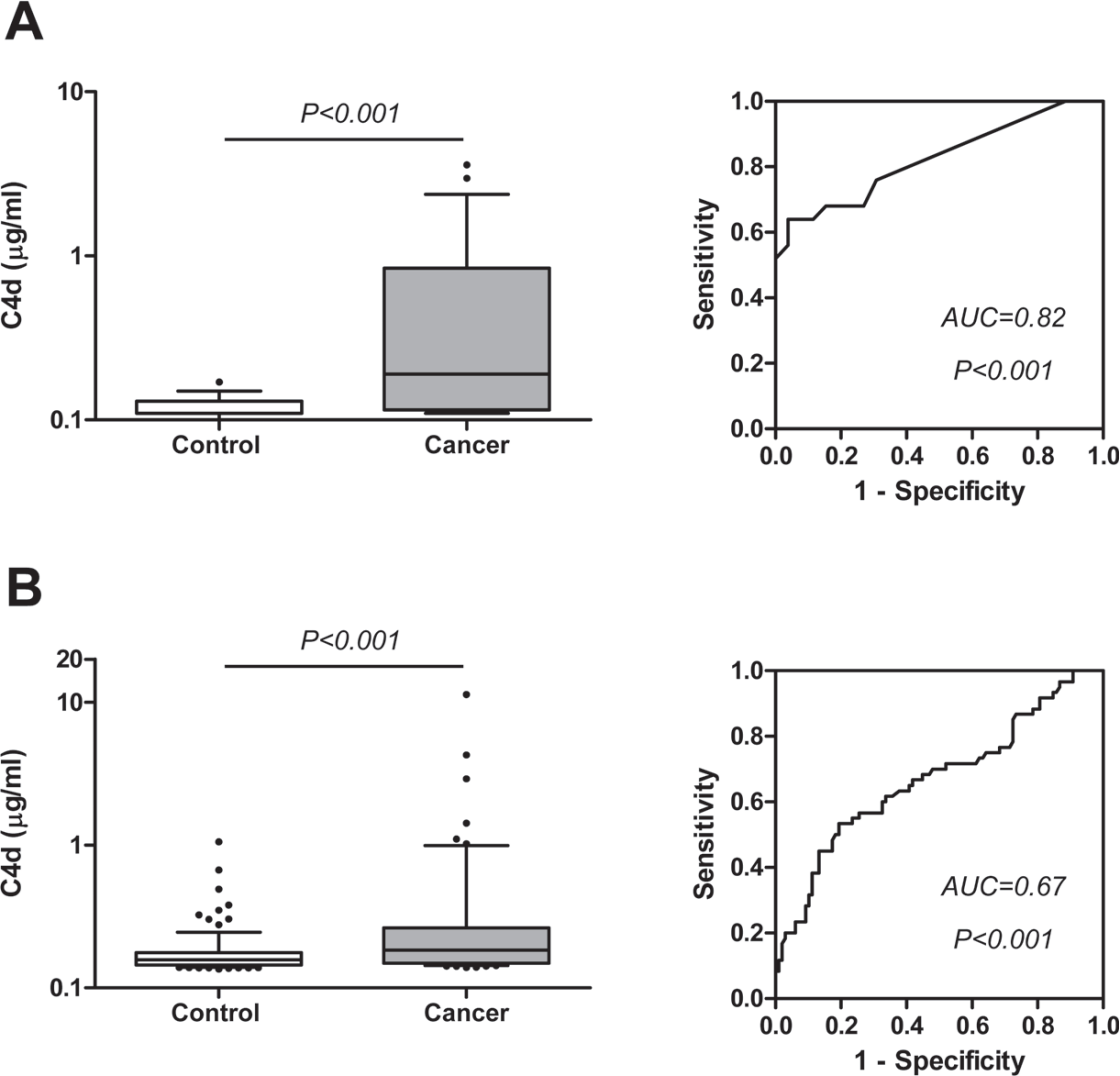
Quantification of the complement component C4d in BAL supernatants from two cohorts (CU and HUVR) of patients with lung cancer and control individuals. A) C4d levels and ROC curve from BAL supernatants of the CU cohort. B) C4d levels and ROC curve from BAL samples of the HUVR cohort. Differences between groups were evaluated using the Mann-Whitney U test. The areas under the ROC curves (AUC) and their associated P values are indicated.

In a previous study we found that the levels of C4d-containing complement degradation fragments (jointly referred as C4d) were increased in BAL samples from lung cancer patients when compared to samples from patients with nonmalignant lung diseases [13]. Briefly, C4d levels, shown as median (interquartile range), were 0.26 (0.11–0.54) μg/ml in the lung cancer group and 0.11 (0.11–0.23) μg/ml in the control group (P<0.001). The area under the ROC curve was 0.73 (95%CI = 0.61–0.84; P = 0.002). This analysis was performed on BAL samples from a cohort of patients herein denoted as CUN cohort. The characteristics of these patients have already been published [15]. In the present study, we sought to validate this result using two independent cohorts of BAL samples: the HUVR and the CU cohorts. In this latter cohort, C4d levels in lung cancer specimens and controls were 0.19 (0.11–0.84) and 0.11 (0.11–0.13) μg/ml, respectively (P<0.001; Fig. 1A). The area under the ROC curve was 0.82 (95%CI = 0.71–0.94; P<0.001). In the HUVR cohort, C4d levels in the cancer group were 0.18 (0.15–0.26) μg/ml, and in the control group were 0.16 (0.14–0.18) μg/ml (P<0.001; Fig. 1B). The area under the ROC curve was 0.67 (95%CI = 0.58–0.76; P<0.001). In this cohort, C4d levels tended to be higher in BAL fluids from small cell lung cancer (SCLC) patients or former smokers, although the differences did not reach statistical significance (Table 1). In accordance with this observation, in our previous study (CUN cohort) we found a significant increase of C4d in former smokers in the cancer group, and a trend towards higher C4d levels in patients with SCLC [13].

### Performance comparison of C4d with other molecular markers

In order to assess the usefulness of C4d as a diagnostic biomarker in BAL fluids, we compared its performance with other potential diagnostic molecular markers. For this purpose, we measured the levels of CYFRA 21-1, C4 and C5a in the CUN, CU, and HUVR cohorts. Except for CYFRA 21-1 and C5a in the CUN cohort, and for C5a in the HUVR cohort, the levels of all the markers were significantly increased in lung cancer patients (Table 2).

**Table 2:** C4d, CYFRA 21-1, C5a, C4, and total protein levels in BAL fluids from individuals of the CUN, CU and HUVR cohorts.

	CUN cohort			CU cohort			HUVR cohort		
	Control (n = 22)	Lung cancer (n = 50)		Control (n = 26)	Lung cancer (n = 25)		Control (n = 98)	Lung cancer (n = 60)	
Marker	Median (IQR)	Median (IQR)	P^1^	Median (IQR)	Median (IQR)	P^1^	Median (IQR)	Median (IQR)	P^1^
C4d (μg/ml)	0.11 (0.11–0.23)	0.26 (0.11–0.54)	<0.001	0.11 (0.11–0.13)	0.19 (0.11–0.84)	<0.001	0.16 (0.14–0.18)	0.18 (0.15–0.26)	<0.001
CYFRA 21–1 (μg/ml)	0.07 (0.02–0.25)	0.09 (0.04–0.19)	0.616	0.02 (0.02–0.03)	0.05 (0.03–0.13)	0.001	0.01 (0–0.02)	0.02 (0.01–0.05)	0.001
C5a (ng/ml)	4.48 (0.63–14.21)	6.26 (2.36–20.09)	0.156	0 (0–0.05)	0.54 (0.04–3.42)	<0.001	0 (0–0.82)	0.16 (0–0.59)	0.580
C4 (μg/ml)	0.87 (0.45–3.24)	3.77 (0.98–6.56)	0.022	0.15 (0.05–0.31)	1.53 (0.50–9.31)	<0.001	0.16 (0.05–0.43)	0.47 (0.09–2.52)	0.001
Total protein (mg/ml)	0.24 (0.12–0.83)	0.74 (0.25–1.43)	0.008	0.14 (0.05–0.41)	0.88 (0.31–1.30)	<0.001	0 (0–0.22)	0.37 (0.19–0.84)	<0.001

^1^Mann-Whitney U test

The accumulation of plasma proteins in the tumor interstitium is a common feature of solid tumors [20]. In fact, plasma proteins can be found in BAL fluids from lung cancer patients [15]. In agreement with this observation, in the three cohorts, the levels of total proteins were found increased in BAL fluids from lung cancer patients as compared to controls (Table 2). To evaluate the possibility that C4d, CYFRA 21-1, C4, and C5a were found in BAL samples as a mere consequence of their extravasation from blood, we analyzed the enrichment of these markers in BAL samples from lung cancer patients with respect to blood using total protein content as reference (Table 3). Very similar proportions of C4 were found in BAL samples and blood, which suggests that the presence of this complement protein in the tumor microenvironment is mainly due to its extravasation. However, CYFRA 21-1, C4d, and C5a showed a markedly higher proportion in BAL fluids, suggesting a local production of these molecules within the tumor microenvironment.

**Table 3:** Enrichment of the markers in BAL fluids from lung cancer patients as compared to their presence in blood.

	Blood		BAL		
	Marker concentration Mean (μg/ml)*	Ratio marker/total protein	Marker concentration Mean (μg/ml)	Ratio marker/total protein	Marker enrichment in BAL**
**CUN cohort**					
Total protein	79000	1	1520	1	1
C4	410	5.2 x 10^−3^	6.04	3.9 x 10^−3^	0.77
C4d	3.5	4.4 x 10^−5^	0.61	4 x 10^−4^	9.06
C5a	0.028	3.5 x 10^−7^	0.012	7.9 x 10^−6^	22.27
CYFRA 21-1	0.013	1.6 x 10^−7^	0.13	8.5 x 10^−5^	520
**CU cohort**					
Total protein	79000	1	870	1	1
C4	410	5.2 x 10^−3^	5.29	6.1 x 10^−3^	1.17
C4d	3.5	4.4 x 10^−5^	0.64	7.3 x 10^−4^	16.60
C5a	0.028	3.5 x 10^−7^	0.0017	1.9 x 10^−6^	5.51
CYFRA 21-1	0.013	1.6 x 10^−7^	0.2	2.3 x 10^−4^	1397
**HUVR cohort**					
Total protein	79000	1	610	1	1
C4	410	5.2 x 10^−3^	2.21	3.6 x 10^−3^	0.70
C4d	3.5	4.4 x 10^−5^	0.57	9.3 x 10^−4^	21.09
C5a	0.028	3.5 x 10^−7^	0.0007	1.1 x 10^−6^	3.24
CYFRA 21-1	0.013	1.6 x 10^−7^	0.07	1.1 x 10^−4^	697

*Average blood concentrations of C4, C4d, C5a, and CYFRA in lung cancer patients were obtained from previous reports [13], [17], [34, 35]. Total protein in blood was calculated in our laboratory using 134 plasma samples from lung cancer patients.

**Calculated as the ratio of the marker in BAL fluids divided by the ratio of the marker in blood.

**Fig 2:**
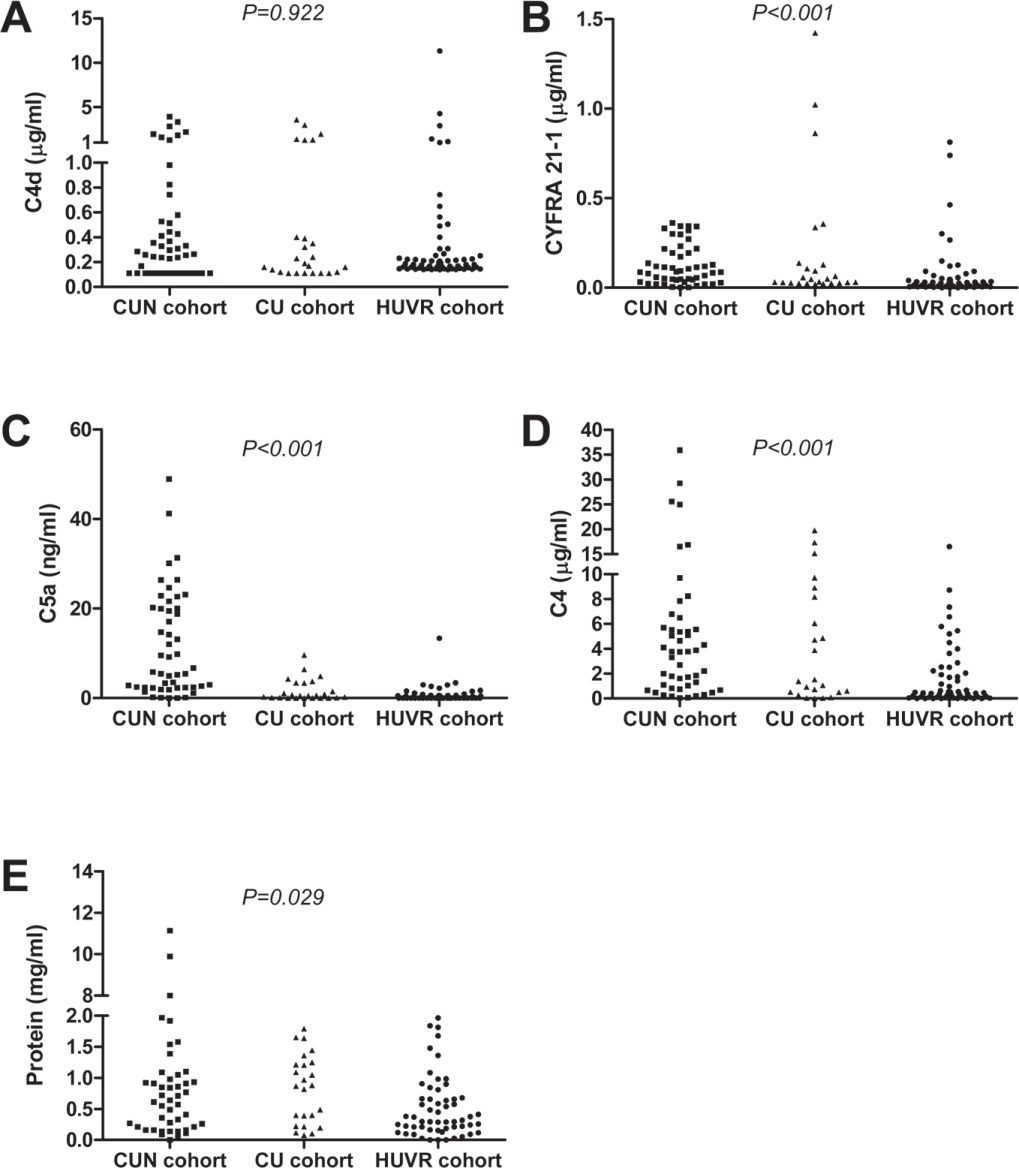
Comparison of the levels of the different markers across the three independent cohorts of BAL supernatants from lung cancer patients. A) C4d (data from the CUN cohort were published previously [13]). B) CYFRA 21–1. C) C5a. D) C4. E) Total protein. P values were calculated using the Kruskal-Wallis test.

A factor that strongly influences the performance and usefulness of a diagnostic marker is its consistency across different patient populations. We therefore compared the levels of the four molecular markers across the three independent cohorts. As shown in Fig. 2, significant differences among lung cancer patients in the CUN, CU, and HUVR cohorts were observed for CYFRA 21–1, C5a, C4, and total protein (P<0.001, P<0.001, P<0.001, and P = 0.029, respectively). We observed higher levels of these markers in the CUN cohort, although the reason for this difference is unclear. Nevertheless, the levels of C4d were remarkably similar across the three series with no significant differences between them (P = 0.922). The consistency of C4d levels allowed us to propose C4d cut-off values with satisfactory sensitivity and specificity across the three cohorts. For example, with a cut-off value of 0.18 μg/ml, the sensitivity in the diagnosis of lung cancer was 62%, 52%, and 53%, with a specificity of 73% (odds ratio (OR) = 4.35, 95%CI = 1.45–13.05, P = 0.009), 100%, and 80% (OR = 4.46, 95%CI = 2.20–9.03, P<0.001) in the CUN, CU, and HUVR cohorts, respectively. When pooling the three studies together, the sensitivity was 56% and the specificity was 82% (OR = 5.94, 95%CI = 3.45–10.24, P<0.001). In summary, we conclude that C4d is produced within the lung tumor microenvironment, resulting in a consistent increase of this molecule in BAL fluids from lung cancer patients.

### Determination of C4d in BAL supernatants as an adjunct to cytology for lung cancer diagnosis

**Fig 3:**
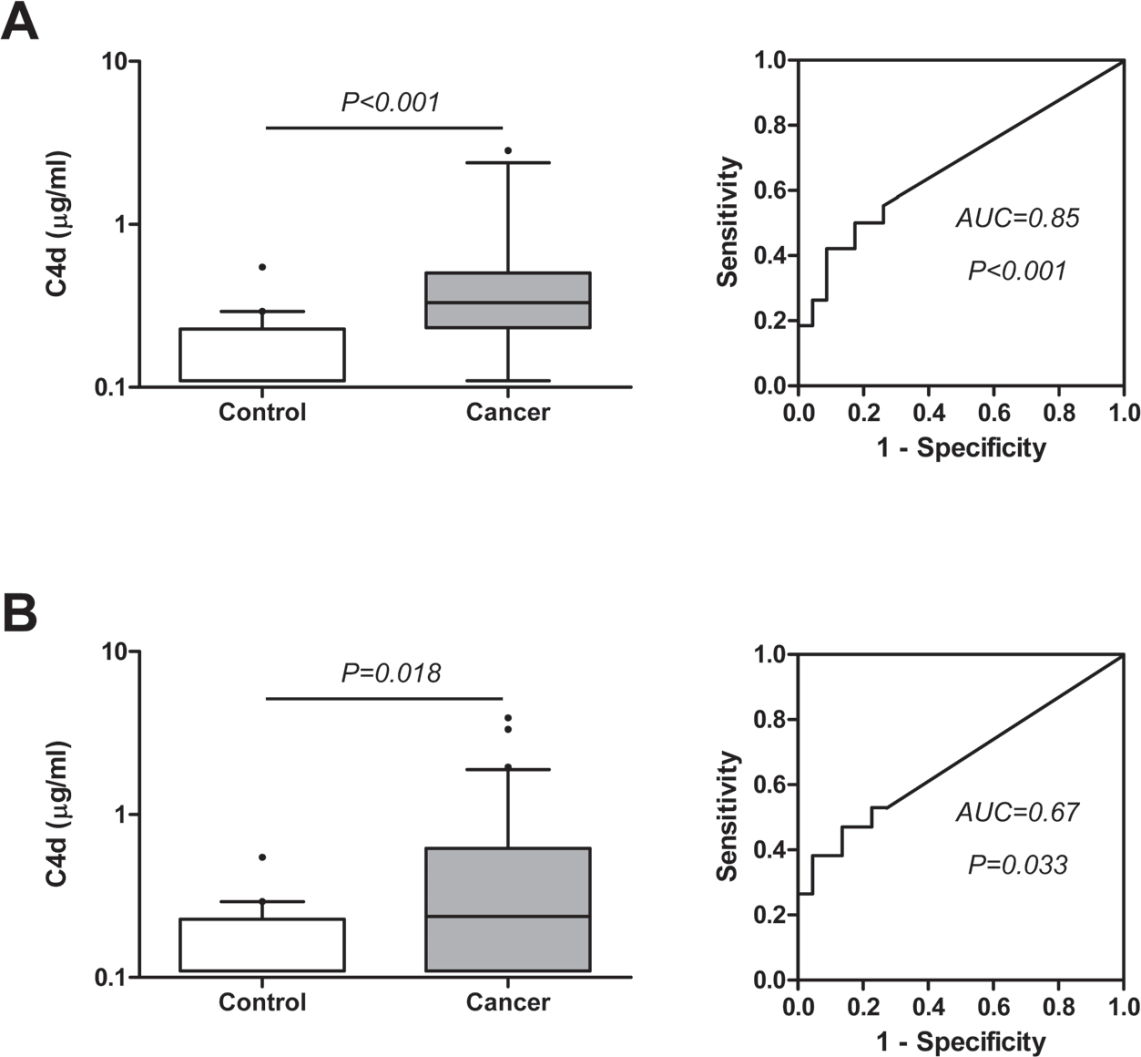
C4d levels and ROC curves from cytologically positive (A) and negative (B) lung cancer BAL supernatants of the CUN cohort. P values were calculated using the Mann-Whitney U test. The areas under the ROC curves (AUC) and their associated P values are indicated.

**Fig 4:**
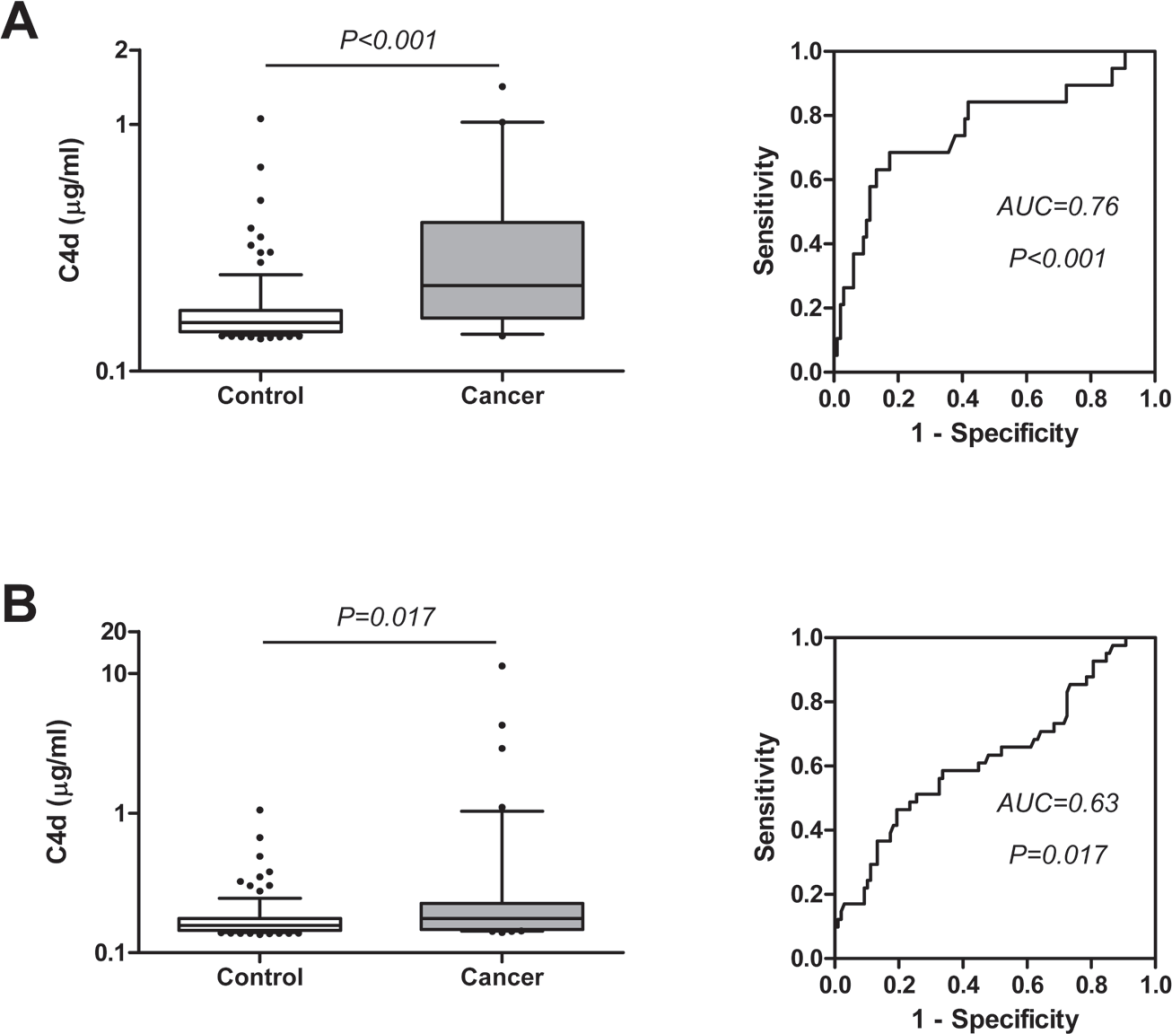
C4d levels and ROC curves from cytologically positive (A) and negative (B) lung cancer BAL supernatants of the HUVR cohort. P values were calculated using the Mann-Whitney U test. The areas under the ROC curves (AUC) and their associated P values are indicated.

We next assessed the capacity of C4d determination to improve the diagnostic performance of the cytological examination of bronchoscopic material, a technique with high specificity but low sensitivity. For this purpose we used the two cohorts from which we had cytology data: CUN and HUVR cohorts. In the CUN cohort, cytological examination of BAL samples yielded a specificity of 100% and a sensitivity of 32%. Examining the diagnostic value of the information provided by C4d, we found that this marker was significantly increased in both cytologically positive and negative BAL supernatants from lung cancer patients (Fig. 3). The levels of C4d in the control group were 0.11 (0.11–0.23) μg/ml and increased to 0.33 (0.23–0.5) μg/ml in cytologically positive samples (P<0.001), and to 0.24 (0.11–0.62) μg/ml in cytologically negative samples (P = 0.018). The areas under the ROC curves were 0.85 (95%CI = 0.71–0.98; P<0.001) and 0.67 (95%CI = 0.53–0.81; P = 0.033), respectively. The diagnostic performance of C4d was better in cytologically positive BAL samples, although no significant differences were found between these two groups (P = 0.187). This result suggests that the determination of C4d levels may be useful even in cases in which the cytological examination of BAL fluids is reported as negative. In fact, using the cut-off value previously established (0.18 μg/ml), 18 out of 34 lung cancers reported as negative by cytological examination were classified as positive by C4d determination, increasing the sensitivity in the diagnosis of lung cancer from 32% to 68%, with a specificity of 73%. This observation was validated with the analysis of the HUVR cohort. Cytological examination in this cohort yielded a sensitivity of 32% and a specificity of 100%. The content of C4d in BAL supernatants significantly increased in both cytologically positive and negative lung cancer BAL supernatants when compared with control subjects (Fig. 4). C4d levels in the control group were 0.16 (0.14–0.18) μg/ml, and increased to 0.22 (0.16–0.40) μg/ml in cytologically positive samples (P<0.001), and to 0.18 (0.15–0.23) μg/ml in cytologically negative samples (P = 0.017). The areas under the ROC curves were 0.76 (95%CI = 0.63–0.88; P<0.001) and 0.63 (95%CI = 0.52–0.74; P = 0.011), respectively. No significant differences in C4d levels were observed between cytologically positive and negative BAL samples (P = 0.123). With a cut-off value of 0.18 μg/ml, 19 out of 41 cancers reported as negative by cytology were classified as positive by C4d, increasing the sensitivity in the diagnosis of lung cancer from 32% to 63%, with a specificity of 80%.

### C4d levels in sputum supernatants from lung cancer patients

**Fig 5:**
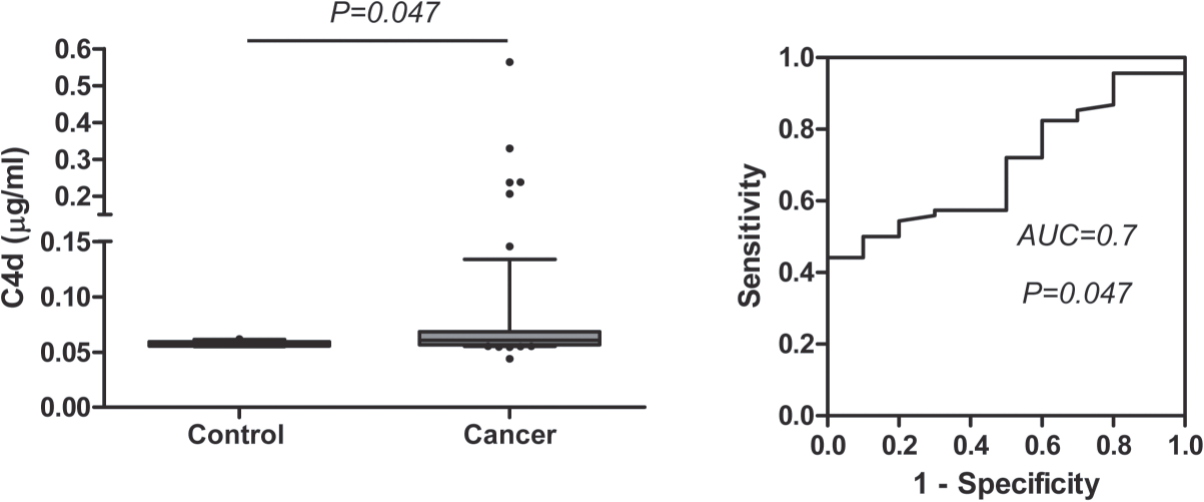
Evaluation of C4d levels in sputum specimens from lung cancer patients and control subjects. P values were calculated using the Mann-Whitney U test. The area under the ROC curve (AUC) and its associated P value are indicated.

Diagnosis of lung cancer by examination of sputum is an attractive alternative to bronchoscopy. In order to preliminary evaluate the utility of C4d as a diagnostic marker in this biological specimen, C4d levels were determined in spontaneous sputum samples from lung cancer patients (n = 68) from the LCCCIO cohort. C4d levels ranged from 0.04 to 0.56 μg/ml with a median (IQR) of 0.06 (0.06–0.07) μg/ml. No significant associations were found between the levels of the marker and clinicopathological data, such as sex, smoking status, histology (NSCLC vs. SCLC) or stage (I-III vs. IV). Finally, we compared the levels of C4d in these sputum samples with the levels in 10 healthy controls. As shown in Fig. 5, C4d levels were slightly higher in sputum supernatants from patients with lung cancer than in those from control subjects (P = 0.047). The area under the ROC curve was 0.7 (95%CI = 0.56–0.83, P = 0.047). With a 0.06 μg/ml cut-off, sensitivity and specificity were 54% and 80%, respectively. In conclusion, C4d levels are significantly higher in spontaneous sputum samples from lung cancer patients than in those samples from cancer-free subjects.

## Discussion

In this study, we demonstrate that C4d-containing complement degradation fragments (jointly referred as C4d) are elevated in bronchial fluids from lung cancer patients and their determination performs better than previously proposed protein biomarkers for lung cancer diagnosis. Our conclusions are supported by four major observations: i) we have validated, in two independent cohorts, the increase in C4d previously observed in a cohort of BAL samples from lung cancer patients [13]; ii) no differences were observed in the levels of the marker across BAL samples from lung cancer patients of three independent cohorts; iii) C4d quantification improved the sensitivity of cytologic examination of BAL fluids; and iv) C4d levels were slightly increased in sputum supernatants from lung cancer patients.

So far, the efforts to identify clinically valuable diagnostic markers for lung cancer have either failed or had limited success. This can be explained in part by the low degree of reproducibility across independent studies due to the high genetic and phenotypic heterogeneity of lung tumors [21]. To overcome this limitation, it has been suggested that immune-related markers might be more homogeneous and sensitive than cancer-derived markers [22]. In this sense, during neoplastic transformation, lung tumor cells from different histological subtypes can be recognized by the complement system [17], [23, 24]. Complement is an important part of the innate immune response that defends the host against non-self elements. Interestingly, due to the elevated expression of complement inhibitors, lung cancer cells control complement activation and are resistant to complement-mediated cytotoxicity [23, 24, 25, 26, 27]. This controlled immune response may result in a permanent release of complement fragments to the tumor microenvironment, which would explain why C4d, a fragment originated from complement C4, is increased in biological fluids from patients with lung cancer [13]. Interestingly, we observed remarkably similar concentrations of C4d across BAL samples from the three studied cohorts. This is a relevant point, since the standardization of measurements between different studies is a major problem in biomarker discovery [28, 29]. In relation to the primary source of C4d, this molecule is a proteolytic fragment of complement C4 generated after activation of the classical complement pathway [16]. Therefore, the most plausible explanation for its presence within the tumor microenvironment would be its local production after activation of this canonical pathway. However, we cannot rule out other mechanisms. For example, exoproteases present in biological fluids from cancer patients can generate peptides from complement proteins [30]. On the other hand, although C4d levels are also increased in plasma from lung cancer patients [13], the levels of the marker in bronchial fluids are noticeably higher than those expected by extravasation, which favors the local origin of C4d.

We also found that C4d performed better than other complement-related markers, C4 and C5a, or the proposed tumor marker CYFRA 21-1. In the case of C4, the precursor molecule for C4d, we conclude that its presence in the airway fluids is mostly due to its extravasation from blood. Similarly, increased levels of other extravasated plasma proteins, such as complement factor H or albumin, were previously observed in bronchial fluids from lung cancer patients [15]. On the other hand, our data suggest a local production of C5a, an anaphylatoxin that can be produced by lung cancer cells [17]. However, this marker was found significantly increased in BAL samples from lung cancer patients in only one of the three cohorts. Similar results were observed with CYFRA 21-1, one of the most studied lung cancer biomarkers in the literature [7, 14]. Our data suggested a local production of CYFRA 21-1 within the tumor microenvironment, but its levels across the cohorts were poorly consistent and differences between malignant and non-malignant samples were not found in one of the series. These observations suggest that C5a and CYFRA 21-1 are produced in the presence of lung tumors, but also in the presence of non-malignant inflammatory conditions common to patients undergoing bronchoscopy.

Given the relatively low sensitivity of the cytological examination of bronchoscopic specimens, more invasive diagnostic tests are usually needed. In this context, a diagnostic procedure useful to determine which patients with suspected lung cancer, but negative bronchoscopies, should undergo additional diagnostic testing would be clinically relevant. In this study, the addition of C4d quantification to the diagnostic procedure increased the sensitivity of bronchoscopy, although reduced its specificity. The reduction in specificity may limit its adoption as a routine clinical diagnostic tool. However, the consistency of the marker across independent cohorts of bronchial fluid samples and its better performance than previously proposed biomarkers encourage the integration of C4d in a panel of biomarkers to complement cytology in the diagnosis of the disease. This is an important point since novel panels of biomarkers such as autoantibodies, microRNAs or proteomic classifiers have yielded promising results in blood specimens from lung cancer clinical cohorts [31, 32, 33]. Moreover, our data suggest that C4d evaluation in non-invasive sputum samples may also be a useful tool for lung cancer diagnosis. This observation, based on a small cohort of sputum samples, merits further evaluation in larger series using more clinically relevant controls with non-malignant lung disease. Other aspects that need to be clarified are the performance of the assay in the different histologic subtypes, as well as the influence of smoking, and specific non-malignant respiratory conditions and treatments on C4d levels. At present, we can conclude that C4d levels are consistently increased in bronchial fluids from lung cancer patients and may complement cytology in the diagnosis of the disease.
